# Ultrasound versus computed tomography guided percutaneous needle biopsy for subpleural pulmonary lesions

**DOI:** 10.3389/fonc.2024.1474531

**Published:** 2024-11-12

**Authors:** Yaoyao Zhou, Xuhong Wang, Rui Han, Shengmin Zhang

**Affiliations:** ^1^ Department of Ultrasound Medicine, The First Affiliated Hospital of Ningbo University, Ningbo, Zhejiang, China; ^2^ Department of Interventional Radiology, The First Affiliated Hospital of Ningbo University, Ningbo, Zhejiang, China

**Keywords:** computed tomography, ultrasound, biopsy, sub-pleural lesions, pulmonary

## Abstract

**Background:**

Sub-pleural pulmonary lesions (SPLs) can be diagnosed by percutaneous needle biopsy (PNB) guided by both computed tomography (CT) and ultrasound (US). This investigation aims to compare the diagnostic accuracy and safety between US- and CT-guided PNB for SPLs.

**Methods:**

This retrospective study analyzed SPL patients who underwent CT- or US-guided PNB in our hospital between January 2022 to January 2023. Furthermore, the technical success rates, duration of procedure, diagnostic yield, diagnostic accuracy, pneumothorax rates, and hemoptysis rates were compared between the 2 groups. Pneumothorax risk factors were assessed via the univariate and multivariate logistic regression tests.

**Results:**

The data indicated that 213 patients who underwent CT- (n = 108) or US-guided (n = 105) PNB diagnosis had SPLs at the final diagnosis. Furthermore, both groups indicated similar operation times (20.1 ± 8.1 min *vs.* 19.9 ± 6.9 min, *p =* 0.793). The diagnostic accuracy and yield of the US group were 100% and 64.8%, respectively, whereas those of the CT group were 99.1% and 72.2%, respectively. Moreover, no significant differences were observed in diagnostic accuracy (*p =* 1.000) and diagnostic yield (*p =* 0.561) between the 2 groups. The CT group indicated markedly higher rates of chest tube insertion (6.5% *vs.* 0.0%, *p =* 0.014) and pneumothorax (24.1% *vs.* 1.9%, *p =* 0.001) than the US group. However, the hemoptysis rates were comparable between the 2 groups (2.7% *vs.* 2.9%, *p =* 1.000). In addition, CT guidance was the independent risk factor of pneumothorax (*p =* 0.003).

**Conclusions:**

In summary, this research indicated that both US- and CT-guided PNB have high diagnostic accuracy for SPLs. However, US guidance may provide better safety than CT guidance.

## Introduction

Peripheral pulmonary lesions are most frequently diagnosed *via* percutaneous needle biopsy (PNB) (1–3). Computed tomography (CT) is employed as guidance equipment because of the spatial resolution. It has been observed that CT-guided PNB is an efficient procedure with a high diagnostic rate (92 - 98%) (4); however, in addition to radiation exposure, it has also been linked with high complication rates. Moreover, 20 - 22% of cases suffer from the incidence of post-procedural pneumothorax (4).

Ultrasound (US)-guided PNB is usually used for diagnosing solid organ lesions, such as in the liver, prostate, thyroid, kidney, and breast (5–7). For pulmonary lesions, US-guided PNB is not commonly used because it is influenced by gas. However, the US-guided PNB can be used for the sub-pleural pulmonary lesions (SPLs) strickly adherent to pleural surface because these lesions are not or slightly influenced by the gas. The US-guided PNB has advantages over CT guidance, such as real-time biopsy needles and target lesion visualization during the procedure and the lack of radiation exposure (8).

Therefore, this investigation aimed to compare the diagnostic accuracy and safety between US- and CT-guided PNB for SPLs.

## Materials and methods

### Study design

This retrospective research was authorized by the ethical board of the First Affiliated Hospital of Ningbo University. Because of the retrospective nature of this study, the requirement of informed consent was waived. This investigation included SPL patients who underwent CT- or US-guided PNB in our hospital between January 2022 to January 2023. The inclusion criteria included patients (a) with SPLs strictly adherent to pleural surface, (b) with solid SPLs, and (c) with a high risk for lung cancer based on clinical/radiology characteristics (3). The exclusion parameters were: (a) patients who underwent PNB previously, (b) SPLs that reduced in size during follow-up, (c) SPLs that were completely blocked by the bone, and (d) patients with chronic cardiac, renal, pulmonary, or coagulatory disorders.

### CT-guided PNB

**Figure 1:**
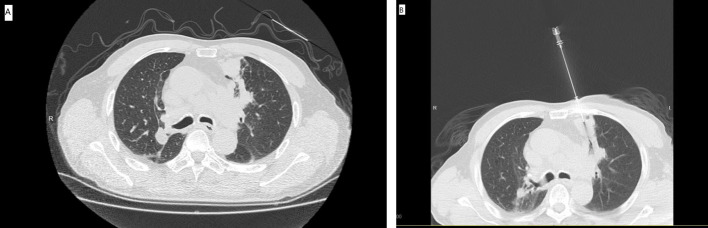
**(A)** The preoperative CT showed the SPL at left upper lobe. **(B)** The CT-guided PNB procedure.

The 16 Slice CT (Siemens, Berlin, Germany) was employed for CT-guided PNB. The CT-guided PNB was performed by 2 radiologists with more than 5 and 10 years of experience for lung biopsy. The set parameters were: 100 mA tube current, 120 kV tube voltage, 0.6 second gantry rotation time, 2 mm thickness, and 1.1 pitch. The patient’s position and needle paths were based on the lesion’s location (**Figure 1A**). During the procedure, the co-axial technique was employed. The lesion samples were harvested by inserting A 17G outer needle (DuoSmart™, Modena, Italy) into the lesions, followed by the insertion of an 18G inner semi-automatic core needle (Wego™, Weihai, China) *via* the outer needle (**Figure 1B**). From each lesion, more than 2 samples were acquired, which were then preserved in 10% formaldehyde for pathological assessment. In addition, the cell blocks were also prepared for cytology assessment. The biopsy procedure-related complications were assessed by a repeat CT scan. The histo-cytological diagnoses were made by two pathologists with 5 and 10 years expertise in lung cancer.

### Ultrasound-guided PNB

**Figure 2:**
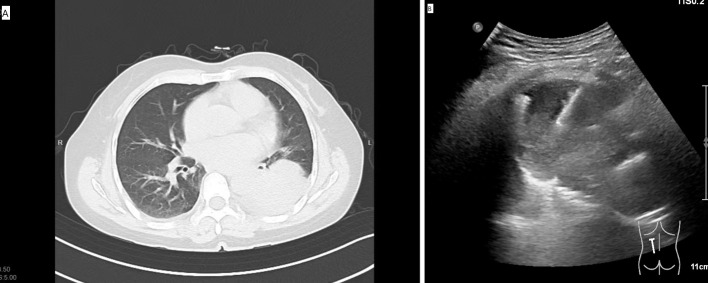
**(A)** The preoperative CT showed the SPL at left lower lobe. **(B)** The US-guided PNB procedure.

For the US-guided PNB, a US system (Philips, EPIQ7) with the convex array probe C5-1 was routinely employed. The US-guided PNB was performed by 2 radiologists with more than 5 and 10 years of experience for lung biopsy. The parameters of the US were: (a) depth: 10-13 cm; (b) time gain compensation: time gain compensation curve is adjusted for the shape of “|” or “”; (c) focus pointe: the focus pointe was set in the midfield zone. The tissue harmonic imaging was activated. The probe was equipped with a holed guide for needle insertion of various angle selections based on the lesions’ size and locations. For the small lesions (1-2 cm), we used a high frequency probe (eL18-4) to guide the PNB because the high frequency probe could provide a high image resolution. The patient’s position was decided in advance *via* the safest and shortest approach to visualize the US probe’s movement (**Figure 2A**). The biopsy needle path toward the lesion was visualized in real time (**Figure 2B**). The PNB procedure was same as the that for the CT group. The biopsy procedure-related complications were assessed by a chest CT scan. The histo-cytological diagnoses were made by two pathologists with 5 and 10 years expertise in lung cancer.

### Assessment

The PNB-based diagnoses can be categorized into 4 types: (a) Suspected malignancy (described as atypical cells suspected of indicated malignancy) (9), (b) Malignancy, (c) Non-specific benignity (defined as benign pathology features that existed through and did not suffice for a formal diagnosis) (9), and (d) Specific benignity (described as lesions that were related to specific infectious diseases or benign tumors) (9). The PNB-based specific benignity and malignancy can be considered as the final diagnoses. However, the PNB-based non-specific benignity and suspected malignancy should be assessed further, such as by repeat biopsy, surgical resection, or CT follow-up. If the lesion size is reduced (≥ 20%) during the follow-up or is maintained (< 20%) for at least 1 year, it could be accepted as benignity (9).

At the final diagnosis, when PNB-based malignancy/suspected malignancy was confirmed as malignant the results were considered true positive (1). Whereas, when PNB-based benignity was confirmed as benignity at the final diagnosis the results were considered true negative (1). Diagnostic accuracy = (true positive + true negative)/all cases. Diagnostic yield = (PNB-based malignancy + PNB-based specific benignity)/all cases.

The PNB-related complications were classified as minor and major complications according to the Society of Cardiovascular & Interventional Radiology guidelines (10).

### Statistical analyses

The not normally distributed and normally distributed continuous data were compared *via* the Mann-Whitney U test and the independent sample t-test, respectively. χ^2^/Fisher exact test was carried out to compare categorical data. Pneumothorax risk factors were assessed *via* univariate and multivariate logistic regression tests. The variables which exhibited a *p-value* < 0.1 in univariate analyses would be included in the multivariate analysis. The subgroup analyses were conducted based on the patients with lung nodules. SPSS 16.0 (SPSS, Chicago, Illinois, USA) was employed for all the statistical measurements and statistically, the *p-value of < 0.05* was deemed as the significance threshold.

## Results

### Patients

This research included 213 patients who underwent CT- (n = 108) or US-guided (n = 105) PNB diagnosis and had SPLs at the final diagnosis (**Table 1**). Furthermore, both groups indicated similar operation times (20.1 ± 8.1 min *vs.* 19.9 ± 6.9 min, *p =* 0.793), respectively.

**Table 1:** Baseline data of the patients.

	CT group (n = 108)	US group (n = 105)	P value
Normal data			
Age (y)	65.1 ± 12.4	67.7 ± 13.0	0.128
Gender (male/female)	72/36	77/28	0.289
Smoking history	18	16	0.776
Emphysema	22	9	0.015
BMI (kg/m^2^)	22.4 ± 3.3	22.3 ± 3.7	0.954
Imaging feature			
Size (mm)	27.8 (Q1: 16.6; Q3: 39.9)	35.5 (Q1: 26.5; Q3: 53.0)	0.001
Lung (left/right)	53/55	46/59	0.441
Lobe (upper/non-upper)	70/38	74/31	0.377
Pleural effusion	25	33	0.189
Biopsy procedure			
Needle- pleura angle (degrees)	65.3 ± 17.9	55.0 ± 13.0	0.001
Prone/Supine/Decubitus	77/27/4	6/76/23	0.001
Number of samples	2.3 ± 0.6	2.6 ± 0.8	0.001
Pneumothorax	26 (24.1%)	2 (1.9%)	0.001
Hemoptysis	3 (2.7%)	3 (2.9%)	1.000
Complications required chest tube	7 (6.5%)	0 (0%)	0.014
Duration of procedure (min)	20.1 ± 8.1	19.9 ± 6.9	0.793

BMI, body mass index; CT, computed tomography; US, ultrasound.

### Diagnostic performance

In the CT group, the PNB-based diagnoses included specific benignity (n = 4), malignancy (n = 74), and non-specific benignity (n = 30). Whereas the final diagnoses included benignity (n = 33) and malignancy (n = 75). **Supplementary Table** indicates the details of the final diagnoses. The diagnostic accuracy and yield were 99.1% and 72.2%, respectively (**Table 2**).

**Table 2:** Diagnostic performance between 2 groups.

	CT group	US group	P value
Technical success rate	100%	100%	
Biopsy pathological diagnosis			0.073
Malignancy	74	63	
Suspected malignancy	0	6	
Specific benign	4	5	
Non-specific benign	30	31	
Final diagnosis			0.561
Malignancy	75	69	
Benign	33	36	
Diagnostic performance			
Diagnostic yield	78/108 (72.2%)	68/105 (64.8%)	0.561
Overall accuracy	107/108 (99.1%)	105/105 (100%)	1.000

CT, computed tomography; US, ultrasound.

In the US group, the PNB-based diagnoses included specific benignity (n = 5), suspected malignancy (n = 6), malignancy (n = 63), and non-specific benignity (n = 31). The final diagnoses included benignity (n = 36) and malignancy (n = 69). **Supplementary Table** indicates the details of the final diagnoses. The diagnostic accuracy and yield were 100% and 64.8%, respectively (**Table 2**).

The rates of diagnostic accuracy and yield were comparable in both groups (*p =* 0.561 and 1.000).

### Complications

In the CT group, 26 patients (24.1%) experienced pneumothorax, while 3 patients (2.7%) suffered hemoptysis. Furthermore, of 26 patients, 7 required chest tube insertion. In the US group, 2 patients (1.9%) experienced pneumothorax, 3 (2.9%) experienced hemoptysis, and none required chest tube insertion. In comparison with the US group, the CT group indicated markedly increased rates of chest tube insertion (*p =* 0.014) and pneumothorax (*p =* 0.001), whereas the hemoptysis rates were comparable in both groups (*p =* 1.000, **Table 1**). In the CT group, 19 patients were classified as minor complication and 7 patients were classified as major complication. In the US group, all of the 5 patients were classified as minor complication.

**Table 3** shows the logistic analyses of pneumothorax. In the univariate analysis, the smaller lesion size (*p* = 0.01), larger needle-pleura angle (*p* = 0.054), decubitus position (*p* = 0.016), and CT guidance (*p* = 0.001). When these factors were included into the multivariate analysis, the independent risk factor was assessed to be CT guidance (*p = 0.003*).

**Table 3:** Predictors of pneumothorax.

Variables	Univariate analysis			Multivariate analysis		
	Hazard ratio	95% CI	P value	Hazard ratio	95% CI	P value
Age	0.999	0.969-1.031	0.959			
Gender						
Male	1					
Female	1.037	0.443-2.431	0.933			
Smoking history	1.596	0.709-3.593	0.258			
Emphysema	1.023	0.424-2.467	0.959			
BMI	1.027	0.917-1.151	0.642			
Tumor size	0.963	0.936-0.991	0.01	0.975	0.947-1.004	0.086
Lung						
Right	1					
Left	0.809	0.363-1.804	0.604			
Lobe						
Non-upper	1					
Upper	1.343	0.596-3.023	0.477			
Pleural effusion	0.678	0.273-1.681	0.401			
Needle- pleura angle	1.026	1.000-1.053	0.054	1.007	0.981-1.033	0.613
Body position						
Prone	1			1		
Supine	0.810	0.326-2.009	0.649	1.475	0.544-4.001	0.445
Decubitus	0.157	0.035-0.703	0.016	1.810	0.217-15.072	0.583
Guidance methods						
CT	1			1		
US	0.061	0.014-0.266	0.001	0.055	0.008-0.382	0.003

BMI, body mass index; CI, confidential interval; CT, computed tomography; US, ultrasound.

### Subgroup analyses

**Table 4** shows the subgroup analyses based on the lung nodules. The results indicated comparable diagnostic yield (62.1% *vs.* 59.0%, *p =* 0.749) and accuracy (100% *vs.* 100%) between CT and US groups. Furthermore, the incident rate of pneumothorax was markedly higher in the CT group than in the US group (24.4% *vs.* 2.6%, *p =* 0.004), while that of the hemoptysis was comparable in both the groups (3.0% *vs.* 5.1%, *p =* 0.627).

**Table 4:** Biopsy procedure and diagnostic accuracy of lung nodules between 2 groups.

	CT group (n = 66)	US group (n = 39)	P value
Biopsy procedure			
Lesion size (mm)	20.1 ± 6.7	21.8 ± 6.0	0.184
Number of samples	2.3 ± 0.6	2.4 ± 0.6	0.407
Duration of procedure (min)	20.9 ± 8.5	18.4 ± 5.4	0.073
Pneumothorax	16 (24.2%)	1 (2.6%)	0.004
Hemoptysis	2 (3.0%)	2 (5.1%)	0.627
Biopsy pathological diagnosis			0.594
Malignancy	37	20	
Suspected malignancy	0	1	
Specific benign	4	3	
Non-specific benign	25	15	
Final diagnosis			0.825
Malignancy	37	21	
Benign	29	18	
Diagnostic performance			
Diagnostic yield	41/66 (62.1%)	23/39 (59.0%)	0.749
Overall accuracy	66/66 (100%)	39/39 (100%)	Not applicable

## Discussion

Currently, PNB is the primary diagnostic procedure for subpleural or peripheral lung lesions. PNB not only differentiates benign and malignant lesions but can also provide samples for molecular analyses, which can guide molecular target therapies (11–13). CT-guided PNB has been used for more than 30 years (14). However, CT-guided PNB has the limitation of lack of real-time monitoring. Some researchers used the C-arm cone-beam CT (CBCT) to achieve real-time guidance during the PNB procedure (15–17). Ren et al. (16) indicated that CBCT-guided PNB has better performance than CT fluoroscopy-guided PNB for small lung lesions radiation dose and complications. However, CBCT radiation affects both patients and operators.

US-guided PNB is also a commonly used method for diagnosing the peripheral lung adherent to pleural surface or pleural lesion without radiation (17–19). Furthermore, US has the advantage of real-time visualization of the target lesion. Jarmakani et al. (17) showed that US guidance could obtain more adequacy sample than CT guidance (98% vs. 87%, P = 0.02). However, Jarmakani study used both fine and core needles and this may cause the bias of the results. Yamamoto et al. (19) found that US guidance could obtain significantly higher diagnostic rate than CT guidance for lesions > 40 mm (94.1% vs. 70.6%, P = 0.009). However, for the smaller lesions (< 40 mm), the US-guided and CT-guided PNB showed similar diagnostic rates (90% vs. 86.8%) (19). However, Yamamoto study contained both pulmonary and pleural lesions.This study compared the clinical effect between US- and CT-guided PNB for SPLs. Compared to the previous studies, the present study only focused on the pulmonary lesions and only used the core needle for biopsy. Both groups indicated high and similar diagnostic yield (72.2% *vs.* 64.8%, *p =* 0.561) and accuracy (99.1% *vs.* 100%, *p =* 1.000). Furthermore, the rates of diagnostic accuracy in US- and CT-guided PNB were more than their previously reported rates (84 - 96%) and (77 - 96%), respectively for SPLs (20–22). The high diagnostic accuracy rates in this study might be because of the co-axial technique employed. The co-axial technique was utilized to acquire adequate samples to achieve a high diagnostic yield and accuracy.

This investigation also indicated notably higher pneumothorax and chest tube insertion rates of the CT group than the US group. Furthermore, the independent risk factor of pneumothorax was identified to be CT guidance. CT-guided PNB is performed blindly, while US-guided PNB of lesions can be performed with real-time visualization. US-guided PNB is advantageous as it can avoid normal lungs from the lesion with respiratory movement by visualization and vessels by color Doppler imaging. Yamamoto et al. (19) reported a substantially higher overall complication rate in the CT group than in the US group (24.3% *vs.* 3.3%, *p < 0.001*). These findings were in consistent with our findings. In addition, they also indicated that the US group pneumothorax rate was 0% (19). Overall, these data suggest that US-guided PNB is a safer technique than CT-guided PNB.

In this study, the CT group showed significantly higher rate of emphysema and smaller lesion size than US group. However, these factors were not associated with pneumothorax after univariate and multivariate logistic regression tests. Similarly, some previous studies also did not show that emphysema and lesion size were associated with pneumothorax (1, 2, 23).

Here, it was found that the hemoptysis incident rates in US- and CT-guided PNB were low and similar (2.7% *vs.* 2.9%, *p =* 1.000). This might be because the included lesions were SPLs and the needle seldom touches the intra-pulmonary vessels during PNB for SPLs.

The subgroup analyses indicated that both US- and CT-guided PNB provided high diagnostic accuracy for sub-pleural lung nodules. However, the US-guided PNB guidance indicated better safety than CT-guided PNB, suggesting that US guidance should be considered initially for performing PNB for sub-pleural lung nodules. In addition, to improve the technical reliability of the US-guided PNB for lung nodules, it is advisable the use of a dedicated probe, US transducers with a central hole for needle passage (24).

However, US-guided PNB for SPLs also has some limitations. Although US has the advantage of real-time visualization of the target lesion, US is not an accurate imaging method for characterizing peripheral lung lesions compared to CT scans. Only peripheral consolidations that are strictly adherent to the parietal pleural surface can be imaged because the interposition of even a few millimeters of air is able to block US signal, thus hiding even very big space-occupying lesions. In addition, the acoustic barrier represented by the bony structures of the thoracic cage reduces the visible pleural surface to 70%. As a result, US cannot replace chest CT in the examination of the whole lung.

There are certain limitations of this study. Firstly, this is a retrospective study; therefore, some baseline data, such as emphysema, lesion size, needle-pleura angle, position, and number of samples, were unbalanced between the 2 groups. Although these data did not interfere with the diagnostic accuracy and complication, the selection bias does exist. Secondly, it is single-center research. Further multi-center, randomized controlled trials are required. Thirdly, US-guided PNB is not suitable for central pulmonary lesions and peripheral lesions not adherent to pleural surface or lesions adherent to pleural surface but covered by the rib cage, therefore, its use is limited.

## Conclusion

In summary, this research indicated that both US- and CT-guided PNB have high diagnostic accuracy for SPLs. However, US guidance may provide better safety than CT guidance.

## Data Availability

The original contributions presented in the study are included in the article/**Supplementary Material**. Further inquiries can be directed to the corresponding author.
