# Supplementary material for: Ultrasound versus computed tomography guided percutaneous needle biopsy for subpleural pulmonary lesions
Source: Front Oncol. 2024 Nov 12;14:1474531. doi: 10.3389/fonc.2024.1474531 (PMC11589378; doi:10.3389/fonc.2024.1474531)
Supplement: Supplementary file 1 [file Table1.doc]

Table Details of the final diagnoses.

|  | CT group | US group |
| --- | --- | --- |
| Malignancy |  |  |
| Adenocarcinoma | 35 | 39 |
| Squamous carcinoma | 26 | 15 |
| Small cell lung cancer | 6 | 5 |
| Metastatic cancer | 2 | 6 |
| Others | 6 | 4 |
| Benign |  |  |
| Inflammatory pseudotumor | 5 | 4 |
| Granuloma | 0 | 4 |
| Tuberculosis | 1 | 4 |
| Mycotic infection | 1 | 2 |
| Diagnostic by the CT follow-up | 24 | 20 |
| Benign tumors | 2 | 2 |

CT: computed tomography; US: ultrasound.
